# Reduction of the Number of Major Representative Allergens: From Clinical Testing to 3-Dimensional Structures

**DOI:** 10.1155/2014/291618

**Published:** 2014-03-23

**Authors:** Ying He, Xueting Liu, Yuyi Huang, Zehong Zou, Huifang Chen, He Lai, Lida Zhang, Qiurong Wu, Junyan Zhang, Shan Wang, Jianguo Zhang, Ailin Tao, Baoqing Sun

**Affiliations:** ^1^Guangdong Provincial Key Laboratory of Allergy & Immunology, The State Key Laboratory of Respiratory Disease, The Second Affiliated Hospital of Guangzhou Medical University, 250 Changgang Road East, Guangzhou 510260, China; ^2^Plant Biotechnology Research Center, School of Agriculture and Biology, Shanghai Jiao Tong University, Shanghai 200030, China; ^3^Guangdong Provincial Key Laboratory of Allergy & Immunology, The State Key Laboratory of Respiratory Disease, The First Affiliated Hospital of Guangzhou Medical University, 151 Yanjiang Road, Guangzhou 510120, China

## Abstract

Vast amounts of allergen sequence data have been accumulated, thus complicating the identification of specific allergenic proteins when performing diagnostic allergy tests and immunotherapy. This study aims to rank the importance/potency of the allergens so as to logically reduce the number of allergens and/or allergenic sources. Meta-analysis of 62 allergenic sources used for intradermal testing on 3,335 allergic patients demonstrated that in southern China, mite, sesame, spiny amaranth, * Pseudomonas aeruginosa*, and house dust account for 88.0% to 100% of the observed positive reactions to the 62 types of allergenic sources tested. The * Kolmogorov-Smironov* Test results of the website-obtained allergen data and allergen family featured peptides suggested that allergen research in laboratories worldwide has been conducted in parallel on many of the same species. The major allergens were reduced to 21 representative allergens, which were further divided into seven structural classes, each of which contains similar structural components. This study therefore has condensed numerous allergenic sources and major allergens into fewer major representative ones, thus allowing for the use of a smaller number of allergens when conducting comprehensive allergen testing and immunotherapy treatments.

## 1. Introduction

Over the years, vast amounts of data regarding worldwide allergy diagnosis and treatment have been accumulated. More recently, allergen researchers have probed this data resulting in the complete cDNA sequencing of a large number of allergens, as well as the determination of their three-dimensional (3D) structure in some cases. However, systematic evaluation of the importance and/or potency of the allergens or their sources has not been investigated thus complicating the identification of specific allergenic proteins when performing diagnostic allergy tests. On the other hand, many patients react to a large number of proteins, and allergic cross-reactivity has been described on many levels [1]. The relatedness of pollen and plant food allergens was recently described based on sequence and/or structure similarity [2, 3], and many kinds of allergens were able to be classified into just a few protein families with a restricted number of biochemical functions [4].

Our previous results showed that 478 allergens retrieved online could be clustered into eight groups, regardless of their biological source [5]. It would be a desirable goal if extensive research on a large number of allergens could be transformed into intensive research on just a few major allergens, hereafter referred to as “representative allergens.” Major allergens are proteins that substantially bind to IgE from more than 50 percent of the patients with that specific allergy [6]. Major allergens have therefore been employed as internal standards in order to standardize allergen vaccines [7]. A detailed characterization of the importance of major allergens and their biological sources would allow for the improvement of allergen standardization and thus help to obtain more effective and safer modalities for the diagnosis and therapy of many allergic diseases.

In the present study, we reviewed our clinical intradermal test (IDT) data from 2001 to 2003 on 62 kinds of allergenic sources and compared the amino acid sequences and 3D structures of the major allergens obtained from different biological sources, with the goal of logically reducing these allergenic sources to a few species, as well as progressively clustering the 280 major allergens obtained from the ExPASy Proteomics Server (June 20, 2012) into several of the most representative major allergens. The results obtained could therefore facilitate more consistent and straightforward allergen research.

## 2. Methods

### 2.1. Allergic Patients and IDT

**Figure 1:**
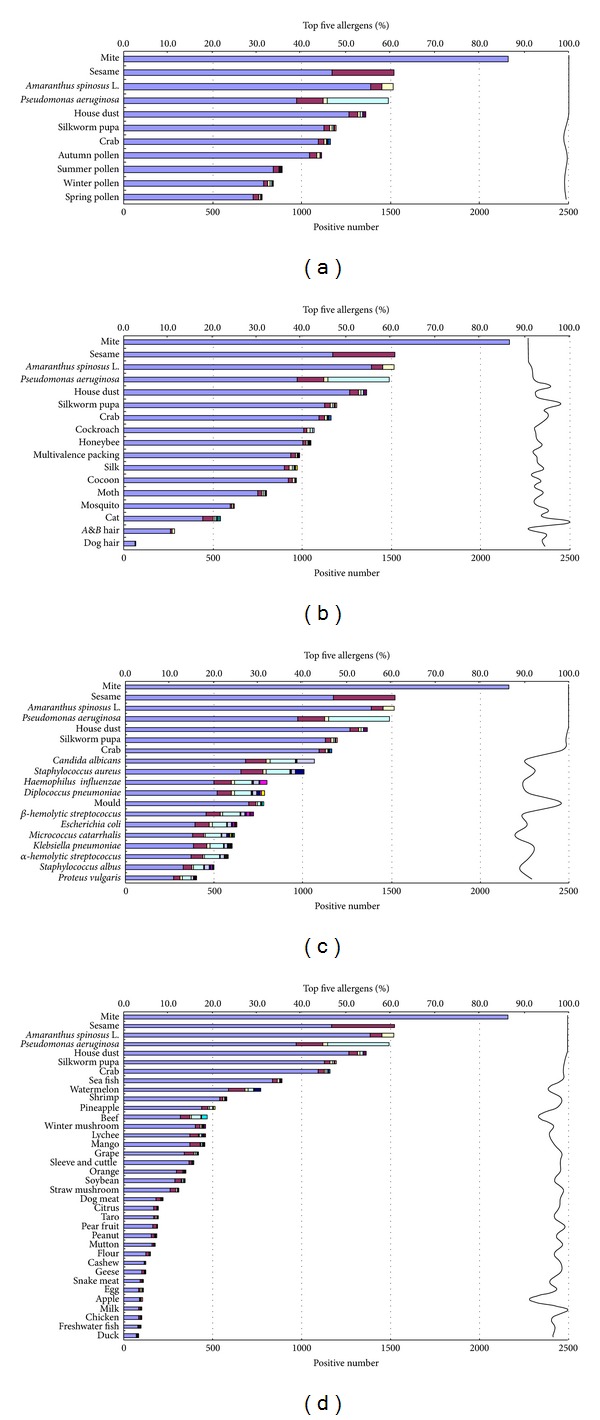
IDT results of 62 allergenic sources on 3,335 patients. The allergenic sources were grouped into 4 panels: (a) pollen; (b) contactant; (c) microbes; (d) food. The top 5 most potent allergenic sources were compared to all other allergenic sources and displayed on each panel. “*A&B hair*” in (b) denotes mix of animal fur and bird feather. The same bar color represents cosensitization among different allergenic sources. And the length of different color bars indicates the proportion of positively sensitized subjects. The different color at the very top of each bar represents the specific positives of each allergenic source. The rightmost curve denotes the total proportion in each allergenic source of the positive cases cosensitized to the top five allergenic sources. Spring pollen is produced by* Acacia confusa* Merr., pine tree, cedar, paper mulberry (*Broussonetia papyrifera*), waxberry (*Morella rubra*), Chinese Mulberry (*Morus australis* Poir.), and Chinese fan palm (*Livistona chinensis* R.). Summer pollen originates from maize, Australian pine (*Casuarina equisetifolia*), Chinaberry (*Melia azedarach*), and* Eucalyptus camaldulensis* Dehnh. Autumn pollen comes from* Mallotus apelta*,* Humulus scandens*, mugwort (*Artemisia vulgaris*),* Vitex negundo*, and* Dioscorea benthamii*. Winter pollen is from cajeput (*Melaleuca leucadendra* L.) and* Bauhinia blakeana* Dunn.

Over the past 32 years, we have intradermally tested (IDT) more than 90,000 patients referred to our allergy clinic. The IDT results of 62 allergen extracts on 3,335 patients in a three-year period (2001–2003) are shown in Figure 1 (children less than three years old were excluded). In our department, IDT has always been exhibited to be effective and safe and was reported to be diagnostically better than the skin prick test [8]. Nevertheless, informed consent for testing was obtained from all patients or their guardians, and the current study was approved by the Ethics Committee of the Second Affiliated Hospital of Guangzhou Medical University.

All allergens extracts were prepared in a sterile environment followed by toxicity and potency evaluation according to an in-house standard protocol as described [9]. After filtration to sterilize, protein levels were quantitated by the Kjeldahl Method [10]. The allergenicity of the extracts was further tested by mice assay to keep the consistency of different batches, followed by the adjustment of stock solution to the standard concentration. All extracts were aliquoted into 10 mL portions at a concentration of 1 : 10 or 1 : 100 and then stored at 4°C until just prior to use.

On the day of the test, the allergen extracts were brought to room temperature and diluted immediately in the solvent (Hengda Pharmacy Co. Ltd, Shanxi, China). Each patient received IDT using the same batch of allergen extracts. IDT was performed as follows: 10–20 *μ*L of each allergen dilution was intradermally injected into each patient with 24–30 allergens tested at a time. Injected allergens were arranged vertically on the upper arm(s) with an interval of 2.5–3.0 cm. A positive control (histamine dihydrochloride, 10 mg/mL) and a negative control (solvent) were also included, and there was no duplicate testing. After 15 min, the size of the wheal was determined by measuring the diameter in two perpendicular directions and then halving the sum. Since bacterial proteins always exhibit a late phase reaction, those results were measured after 24 hrs. The reaction was regarded as positive if the calculated wheal diameter was more than 5 mm.

### 2.2. Relationship of the Allergenic Sources

To assess whether the distribution of positive reactions to each allergenic source reflects a correlation between different allergenic sources, the number of patients with positive IDT reactions to each allergenic source was counted. Patients showing multiple positive reactions to different allergenic sources were counted once for each individual allergenic source they reacted to. All the allergenic sources were ranked according to patient counts, since there may be cosensitization to different allergenic sources.

### 2.3. Overall Distribution of the Allergens Worldwide

The nonredundant allergen data were extracted from the IUIS allergen list (June 20, 2012) available at the website http://www.allergen.org/. The overall research status of allergens worldwide was analyzed as follows: the number of allergenic species and the related allergen numbers within each taxonomic category were recorded. The consistency of the two distributions thereof was investigated by* Kolmogorov*-*Smirnov test*, which measures the maximum difference between two cumulative distribution functions and calculates the probability that the two observed distributions would exhibit a difference at least that large if the samples were drawn from identical populations [11]. The parameters tested are as follows: *D*_*n*,*n*1~*n*2_ = Maximum  (*S*_*n*1_ | *x* | −*S*_*n*2_ | *x*|), $D_{n,0.01}=1.63/\sqrt{n}$, $D_{n,0.05}=1.36/\sqrt{n}$, $D_{n,0.10}=1.23/\sqrt{n}$, *n* = *n*_1_∗*n*_2_/(*n*_1_ + *n*_2_), where *n*_1_ and *n*_2_ denote the number of allergenic species and the number of allergenic proteins within each taxonomic category, respectively.

### 2.4. Clustering of Major Allergens by Amino Acid Sequences

Another set of data related to the amino acid sequences of major allergens was retrieved on June 20, 2012, by searching UniProtKB/Swiss-Prot (http://www.expasy.org/) using the keyword “major allergen” and was selected from the IUIS allergen list (http://www.allergen.org/) by IgE-binding potency. The phylogenetic relationship among the major allergens was inferred by the free package Clustal W 1.83 [12] and MEGA5.0 [13] using the alignments of the amino acid sequences. In the output tree, each single, line-linked, large, and dense group of allergens was taken as one cluster or subcluster. The uppermost allergen sequence in each large and dense cluster in the output tree was retained and taken as the core sequence, with other sequences eliminated. Progressive clustering was repeated by manual iterative selection and alignment of the core sequences. The alignment cycle was stopped when any two allergens were no longer able to be clustered into one subcluster. These allergens are hereafter referred to as “major representative allergens.”

The same clustering procedure above mentioned was applied on the corresponding allergens in which Allergen Family Featured Peptides (AFFPs) are located. AFFPs are allergen-specific peptides panned from nonredundant allergens and harbor perfect information with noise fragments eliminated because of their similarity with nonallergens. 534 AFFPs can correctly discriminate 2290 allergens at the highest sensitivity and specificity and make the underlying software SORTALLER outperform other methods at present [14], which demonstrates that 534 AFFPs have a powerful representativeness.

### 2.5. Comparison of the 3D Structures of the Major Representative Allergens

To compare the three-dimensional (3D) structures of the major representative allergens, the 3D structure of each major representative allergen was modeled in SWISS-MODEL workspace, a web-based integrated service dedicated to protein structure homology modeling and assessment that can be accessible via the ExPASy Bioinformatics Resource Portal [15]. The modeling results were viewed in SWISS-PdbViewer v3.7, an integrated sequence-to-structure workbench [16], followed by adjusting the presenting orientation to facilitate the comparison of the 3D structures of the different major representative allergens.

## 3. Results

### 3.1. Distribution of Positive Reactions to Allergenic Sources in Southern China

Intradermal tests using 62 allergenic sources were performed on 3,335 allergic patients. 3,084 of these patients reacted to at least one of the allergenic sources, with the positive frequency being 89.8%. All the allergenic sources used were aligned according to the number of patients with positive reactions. For convenience, the 62 allergenic sources were divided into four general groups, that is, contact allergens, food allergens, pollen allergens, and microbial allergens. The top seven most potent allergenic sources across all groups were then analyzed as to the frequency of their positive reactions when compared to the positive reactions seen with the specific allergens in any particular group. As shown in Figure 1, different allergenic sources show different degrees of positive reactions. Mites (*Dermatophagoides pteronyssinus* and* D. farinae*) were the number one allergenic source, resulting in more than 70% of the positive reactions among all the four groups. Reaction to sesame (*Sesamum indicum* L.) ranked second with 49.3% of the positive reactions. Spiny amaranth (*Amaranthus spinosus* L.),* Pseudomonas aeruginosa*, and house dust were next with 49.2%, 48.3%, and 44.2% of the positive reactions, respectively. In total, these five allergenic sources express 88.0% to 100% of the positive reactions when compared with microbial allergens, 90.6% to 100% when compared with food allergens, 96.3% to 100% when compared with contact allergens, and 99% to 100% when compared with pollen allergens. That is to say, these five allergenic sources are the most potent and show the most reactivity among the patients we tested; hence we have designated them as the major representative allergenic sources.

### 3.2. Species Distribution of Allergens Studied Worldwide

**Figure 2:**
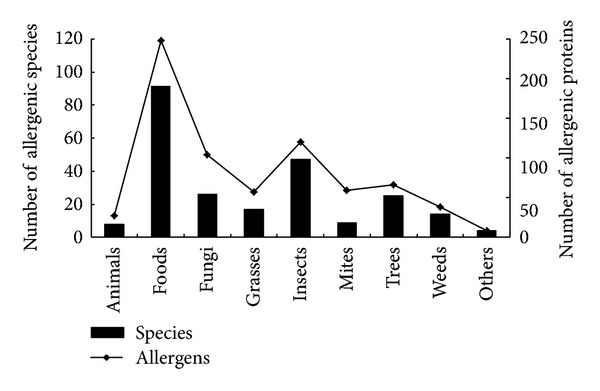
The consistency of two distributions: allergen related species distribution (■) and the distribution of the number of allergenic proteins within each species (▲). The allergens data were downloaded from the IUIS website (http://www.allergen.org/) on June 20, 2012. Estimated parameter for* Kolmogorov*-*Smirnov test* is 0.06642, less than the threshold values (*D*_*n*,0.01_ = 0.1212, *D*_*n*,0.05_ = 0.1011), thus statistically validating the consistency of the two distributions.

At the date of data retrieval for this study (June 20, 2012), 727 nonredundant allergens were listed in the allergen website (http://www.allergen.org/), relating nine categories and 241 species altogether (Figure 2). In terms of categories, foods contain the highest number of allergenic species with insects and fungi being the next most frequent. For allergenic proteins within each category, foods are also number one with fungus and insect allergens ranking second and third. Animals, weeds, and grasses possess the least number of species. In terms of species, the most abundant allergens are possessed by ragweed, timothy, olive, mite, cat,* Aspergillus fumigatus*, peanut, latex, and so forth. The number of allergenic species and the number of allergens in each category constitute two distributions (Figure 2).* Kolmogorov*-*Smirnov test* shows no significant statistical difference (*P* < 0.05) between the number of allergenic species in each category and the number of allergens in each category (Table 1). It indicates that the two distributions were drawn from an identical population, and that the number of allergens is closely related to the number of allergenic species. That is to say, in terms of the allergen research realm worldwide, allergens evolved in parallel from one species to another; no emphasis was prescribed to a certain species/allergen.

### 3.3. Progressive Clustering of the Major Allergens and AFFPs

**Figure 3:**
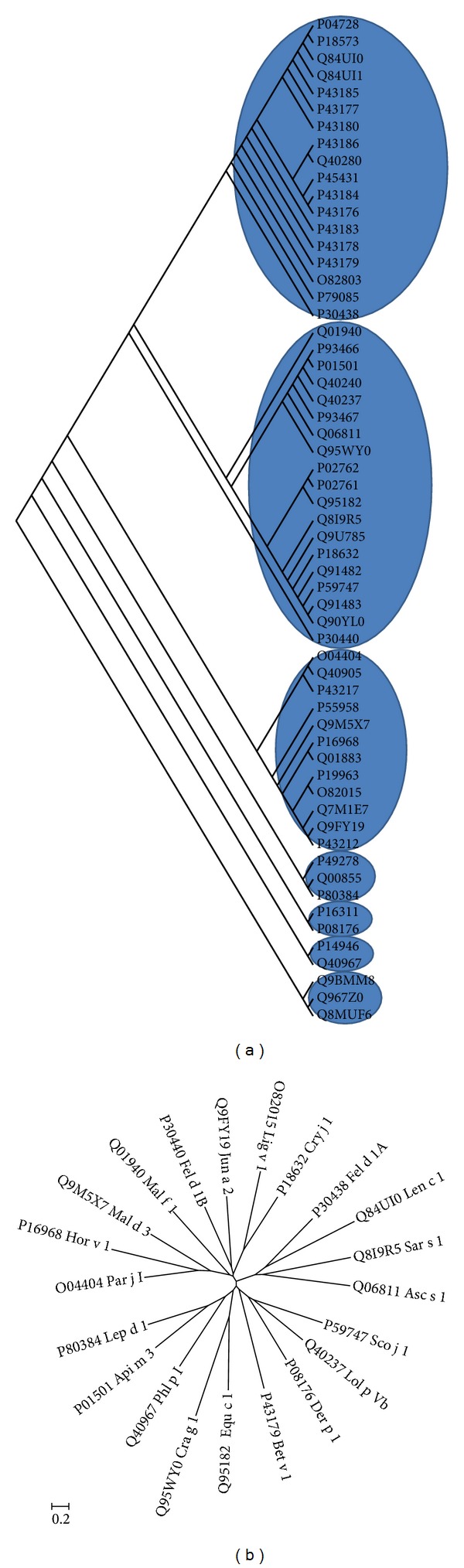
Progressive clustering of the major allergens acquired in UniProtKB/Swiss-Prot database. (a) Phylogeny tree of 59 major allergens constructed and tested by Maximum Liklihood from 280 major allergens. (b) Bootstrap consensus tree of 21 major representative allergens reduced from 59 major allergens.

Previously, we retrieved online 478 allergen sequences and clustered them into eight groups by sequence similarity [5]. In this study, we focused on major allergens and retrieved 280 entries, and 59 major allergens were retained after initial reduction. Clustering results showed that the 59 major allergens were initially classified into seven clusters (Figure 3(a)). Two or more neighboring clusters were combined to form a new data source for further clustering. This procedure was iterated until the last clustering exhibited 21 allergens that were distantly related to each other (Figure 3(b)). Further alignment showed that several pairs of allergens could be respectively grouped together, but with less than 15% pairwise positives in amino acid sequences and most of them exhibited different tertiary structures (See next part). Therefore, no further clustering was assigned to these allergens, and tertiary structure analysis was performed on the identified 21 major allergens.

**Figure 4:**
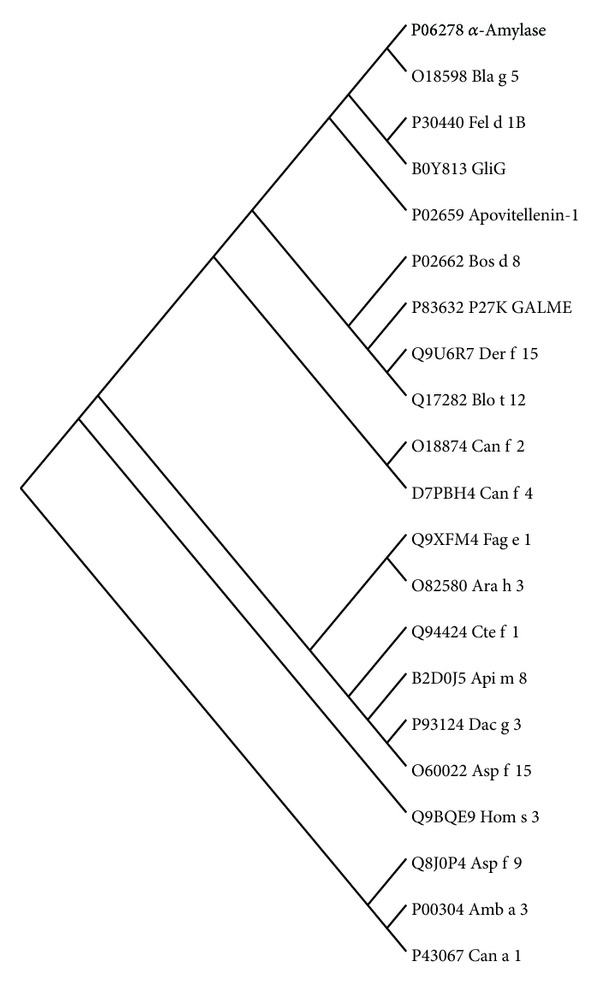
Maximum Parsimony Tree of 21 representative allergens reduced from the allergens corresponding to 534 AFFPs.

Groups with remote homology (<20%–35% in local region) were represented by single entries. This method allowed us to reduce 534 AFFPs into 21 allergens (Figure 4) through five cycles of “cluster-selection-alignment” step, the same as that for major allergens. All the core peptides contain 3–5 matching residues with adjacent mismatches.

### 3.4. Overall Structure Description of the 21 Major Representative Allergens

**Figure 5:**
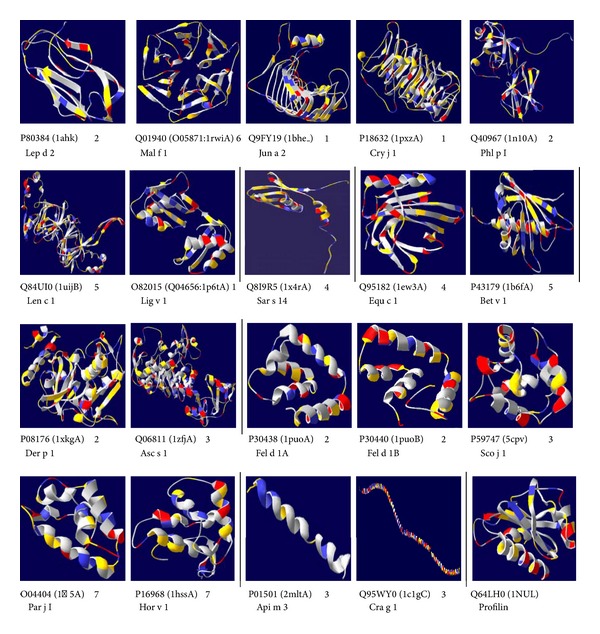
Ribbon diagrams of profilin (Q64LH0) and 19 out of the 21 major representative allergens subtracted. The 7 structural classes are separated by vertical lines. PDB codes are shown in brackets; the following numbers indicate the initial sequence clustering groups. Q64LH0 was not retrieved as a major allergen thus without initial cluster number. Q9M5X7 and Q40237 were omitted for space consideration.

3D structure modeling was completed for most of the above 21 allergens by an automated mode or template identification mode in a SWISS-MODEL workspace [15]. Only O82015 [17], homologous to olive allergen Ole e 1 [18], and Q01940, a major allergen Mal f 1 from* Malassezia furfur* [19], had no identifiable protein structures by a SWISS-MODEL search. Therefore, their 3D structures were modeled by homology to related proteins from the nearby superfamilies Q04656 [20] and O05871 [21]. The 3D structures of the 21 major representative allergens are depicted in Figure 5.

When inspected from spatial structural orientations and surface exposures of the allergens, all of the 21 allergens were shuffled against the initial clustering and interestingly fell into seven structural classes (Figure 5). However, this classification is complicated by the existence of similar structural scenarios in different structural classes.

- Up-and-down *β*-barrel: includes P80384, the major allergen Lep d 2 from Fodder mite (*Lepidoglyphus destructor*) [22], as well as other group 2 allergens from dust mite species, for example, Der f 2 (P49278) from* Dermatophagoides farinae* [23].
- *β*-meander and/or *ψ*-loop constituted calyx, shortened as *β*(*α*) calyx, such as hexagon, cradle, and globose twins: includes allergens Q01940 [19], Q9FY19 [24], P18632 [25], Q40967 [26], Q84 UI0 [27], and O82015 [17].
- *α*-*β* structured crane: includes Q8I9R5, an allergen from* Sarcoptes scabiei* type hominis [28].
- *α*-*β* arranged banana string: includes Q95182 [29] and P43179 [30].
- *α*(*β*) formed complex: includes P08176 [31] and Q06811 [32].
- *α*-helix built clips: a large group including P30438, P30440, P59747 [33], Q40237 [34], O04404 [35], Q9M5X7, and P16968 [36]. Interestingly, P30438 and P30440, chain 1 and chain 2 of cat allergen Fel d 1 [37], configure a pair of chiral molecules on the 3D level. O04404, Q9M5X7, and P16968, originating from different taxonomic species but classified into the same initial Cluster 7, here exhibited structures similar to each other.
- *α*-helix spiral cord: includes P01501 [38] and Q95WY0 [39], both of which came from the same initial cluster III. P01501 is an allergen Api m 3 from honeybee (*Apis mellifera*), also a main toxin of bee venom with strong hemolytic activity. Q95WY0 is the major oyster allergen and tropomyosin from the pacific oyster* Crassostrea gigas*. These two allergens exhibited similar structures but displayed different lengths of their spiral cord.

## 4. Discussion

There is a tight link between allergen diagnosis and immunotherapy. After obtaining the results of allergen intradermal testing, specific IgE diagnosis, and/or even challenge assays, those allergen(s) with the highest positive scores would typically be chosen for use in immunotherapy on the allergic patients. The advent of molecular biology and bioinformatics heralded an unprecedented breakthrough in the development of recombinant allergens engineered to have the same immunological characteristics as natural allergens. Many studies have therefore used recombinant allergens in place of their natural counterparts. Unfortunately, allergen screening from one species to another in parallel only displays an ostensible prosperity of allergen study and could not pinpoint the importance of each allergen. The accumulation of a large quantity of overlapping data is threatening to undermine the achievements of allergen research. Whereto allergen research goes becomes a compelling question.

Facing this question, we firstly cast a meta-analysis on 62 allergenic sources used for intradermal testing on 3,335 patients in a three-year period. The result demonstrated that 88% to 100% of the patients were cosensitized to the top five allergenic sources and assumed that these five allergenic sources would have positive immunotherapeutic effects on the majority of the patients and that the remaining allergenic sources would have minor effects on the patients when used for immunotherapy. All these data corroborate that mite, sesame (*Sesamum indicum* L.), spiny amaranth (*Amaranthus spinosus* L.),* Pseudomonas aeruginosa*, and house dust are the five most prevalent allergenic sources and can well represent the 62 allergenic sources identified in southern China.

Basically, each extract from a single individual allergenic source is a mixture containing about ten allergenic proteins. Certainly, house dust is a mixture as well and contains different allergenic proteins. Hence, using house dust for diagnosis is similar to general screening of allergies with allergen mixes, such as fx1, fx5, mx1, and Phadiatop of UniCAP [40]. The allergens in the top five allergenic sources hence constitute a potent and limited allergen aggregate. Besides the cross-reactivity among some allergens thereof, as deduced from the cosensitization to different allergenic sources, what their relationship is in the repertoire becomes an urgent question. Our unpublished clinical data demonstrated that the allergen preparations from either* D. pteronyssinus* or* D. farinae* can be administered to patients allergic to either mite source and can achieve similar immunotherapeutic effects, which suggests the mutual substitution of the two allergens. This result, corroborating Weber's summary [41], also suggests that it is possible to allow the substitution of the closely related allergenic sources by major representative ones. It is tempting to further think that allergens existing in the five major allergenic sources can be reduced to fewer nonredundant and nonhomologous ones. Hence, it is crucial to converge efforts on the typical representative allergens for further research.

A systematic classification of all allergens by protein taxonomic family and even by structure has long been needed. A former study found that only 52 motifs matched 644 of 779 allergen sequences from all types of sources [42]. Mueller and colleagues argued that primary sequence comparisons could sometimes miss conserved elements of a protein, which can only be seen at the structural level, and that a comparative structural modeling approach could reveal these structural similarities undetectable at the sequence level [43]. Based on these results, the present study focused on major allergens over general ones and performed progressive clustering and manual subtraction of sequence redundancy of major allergens. Twenty-one major representative allergens were subsequently retained and were further classified into seven structural patterns, with many allergens from different sequence groups compiled in one structural class, thus validating the limitation of sequence comparison [44]. Structure class VI, for example, includes contact allergens, pollen allergens, and food allergens. Although they have low sequence homology, these allergens share similar structure scenarios with each other. Moreover, we also found that panallergen profilin (e.g., Q64LH0 [5]) exhibits a configuration of *α*-*β*-*α* layers (Figure 5) and is analogous to 2 EF-hand configuration (polcalcin, e.g., P59747 [33]) and even more similar if the mesial layer is replaced by an *α* helix-formed clip. Further analysis showed that different kinds of allergens, no matter whether they are in the same structure class or not, would share similar structure scenarios in part of their component elements. These results not only theoretically confirmed the clinical relevance between profilin and polcalcin [45], but also suggested the relevant relationship between 2 EF-hand calcium-binding proteins (P59747 [33]) and Poa p IX/Phl p VI allergen family (Q40237 [34]), cereal trypsin/alpha-amylase inhibitor family (P16968 [36]), nonspecific lipid-transfer protein (O04404 [35]), and even uteroglobin (P30438 [37]) and Ole e 1 (O82015 [17]). Major allergen Ole e 1, for example, also harbors a profilin structure-like component element (Figure 5), suggesting its panallergen characteristics [18]. All the results mentioned above are theoretically supported by remote homology modeling and protein profile comparison [46, 47] and thus have drawn a picture of a cross-reactivity network among taxonomically different allergens and even allergens with nil or low sequence similarity and explained the underlying basis of the universal existence of cosensitization to different allergenic sources by individual patients disclosed by the present study.

The 3D structures of a continuously increasing number of allergens are currently being solved. Therefore, it is possible to produce recombinant allergens that exactly mimic their natural wild types and even to produce genetically engineered hypoallergens with nil or low IgE reactivity but retained T-cell reactivity. As the present study demonstrates, clinical cosensitization to multiple allergenic sources can be attributed to a few major allergenic sources; major allergens from different species can be logically reduced to 21 major representative allergens and even grouped into 7 or less structure classes with similar structure scenarios shared by different allergens. It is tempting to think that there might not be a need to unequivocally search all the undeveloped species for totally novel allergen genes or to equivalently test numerous allergenic sources on patients. Thus, it is cost-efficient, practical, and crucial to have the recombinant allergen research focused on the major representative allergens or core AFFPs for immunotherapy strategy development and diagnosis formulation.

We recently noticed that single or fewer major allergens can not only be used to diagnose the genuine sensitization of patients to a given allergen or to the cross-sensitization to several allergenic sources, but also be used for allergen-specific immunotherapy to yield the same effects as the whole allergen mixtures in allergic patients [14, 48, 49]. Intensive clinical evidence has also proven that specific immunotherapy with one kind of allergenic reagent can prevent both the progression of allergies and the acquisition of new allergic sensitizations [50]. All these conclusions corroborate our results.

## Figures and Tables

**Table 1:** Parameter estimate for *Kolmogorov*-*Smirnov test *(Complementary for Figure 2)*.

Group	Frequence		Cumulate Frequence		Cumulate Frequency		*S*_*n*1_ | *x* | −*S*_*n*2_ | *x*|
	Species no.	Allergens no.	Species no.	Allergens no.	Species no.	Allergens no.	
Others	4	8	4	8	0.01660	0.01100	−0.00560
Animals	8	27	12	35	0.04979	0.04814	−0.00165
Mites	9	59	21	94	0.08714	0.12930	0.04216
Weeds	14	38	35	132	0.14523	0.18157	0.03634
Grasses	17	57	52	189	0.21577	0.25997	0.04420
Trees	25	66	77	255	0.31950	0.35076	0.03126
Fungi	26	104	103	359	0.42739	0.49381	**0.06642**
Insects	47	120	150	479	0.62241	0.65887	0.03646
Foods	91	248	241	727	1	1	0

*The allergen data analyzed were downloaded from the IUIS website (http://www.allergen.org/) on June 20, 2012. *D*_*n*,241~727_ = Maximum (*S*_*n*1_ | *x* | −*S*_*n*2_ | *x*|) = 0.06642, while the threshold values were *D*_*n*,0.01_ = 0.1212, *D*_*n*,0.05_ = 0.1011.
